# Digital Twin-Based Virtual Reality Framework for Interaction and AI-Assisted Control of a Parallel Surgical Robot

**DOI:** 10.3390/s26144410

**Published:** 2026-07-11

**Authors:** Florin Covaciu, Nadim Al Hajjar, Anca-Elena Iordan, Radu Corina, Bogdan Gherman, Andrei Cailean, Andra Ciocan, Alexandru Pusca, Paul Tucan, Doina Pisla

**Affiliations:** 1 Research Center for Industrial Robots Simulation and Testing—CESTER, Technical University of Cluj-Napoca, 400114 Cluj-Napoca, Romania; florin.covaciu@muri.utcluj.ro (F.C.); andrei.cailean@mep.utcluj.ro (A.C.); alexandru.pusca@mep.utcluj.ro (A.P.); paul.tucan@mep.utcluj.ro (P.T.); 2 European University of Technology, European Union; anca.iordan@cs.utcluj.ro; 3 Department of Surgery, “Iuliu Hatieganu” University of Medicine and Pharmacy, 400347 Cluj-Napoca, Romania; nadim.alhajjar@umfcluj.ro (N.A.H.); andra.ciocan@umfcluj.ro (A.C.); 4 Department of Computer Science, Technical University of Cluj-Napoca, 400027 Cluj-Napoca, Romania; 5 Department of Internal Medicine, “Iuliu Hatieganu” University of Medicine and Pharmacy, 400347 Cluj-Napoca, Romania; corina.radu@umfcluj.ro; 6 Technical Sciences Academy of Romania, B-dul Dacia, 26, 030167 Bucharest, Romania

**Keywords:** virtual reality, parallel robot, surgical robot, simulator training, VR headset, artificial intelligence, user interface

## Abstract

The rapid advancement of robot-assisted minimally invasive surgery (RAMIS) has created an increasing demand for integrated solutions that combine advanced robotic actuation, sensing, and intelligent control within unified training and operational frameworks. This paper presents a Digital Twin–based virtual reality (VR) interaction and control system developed for an innovative parallel surgical robot, designed to support both surgical training and real-time robot interaction. The proposed framework extends a conventional VR simulator into a bidirectional Digital Twin architecture, enabling real-time synchronization between a virtual environment and the physical robotic system. The system integrates the ATHENA parallel robot, characterized by a 4-degree-of-freedom architecture with a Remote Center of Motion (RCM) constraint, together with a flexible laparoscopic instrument providing enhanced dexterity. Interaction is achieved using VR controllers, allowing intuitive manipulation of the robotic system within an immersive environment. To enhance operational performance, an artificial intelligence module based on neural networks is integrated as an assistive component, providing real-time trajectory refinement and motion guidance. The trained model is deployed using an ONNX-compatible runtime, ensuring efficient inference and seamless integration within the control architecture. The proposed system is validated through experimental evaluation of user interaction and task execution performance, as well as through external motion assessment using an OptiTrack optical tracking system. The results demonstrate improvements in motion stability, execution efficiency, and user interaction quality, while maintaining a high level of control intuitiveness. The findings highlight the potential of Digital Twin–based VR systems as a unifying platform for surgical training, interaction, and intelligent assistance in next-generation medical robotic systems.

## 1. Introduction

Simulation has become an essential component of modern surgical training because it enables repetitive practice in a safe, controlled environment whose complexity can be adapted to the trainee’s skill level. The acquisition of surgical skills should begin in simulated settings before entering the operating room to improve procedural outcomes and patient safety [1]. In this context, virtual reality (VR) provides the possibility of creating complex and interactive digital environments in which users can explore, learn, and practice realistic scenarios under controlled conditions [2,3]. Owing to continuous advances in processing power, device miniaturization, and rendering quality, VR technologies have evolved from gaming-oriented platforms into valuable tools for education, training, and healthcare [4,5,6]. Previous studies have shown that VR simulators can significantly improve surgical performance by reducing errors, accelerating skill acquisition, and enhancing procedural efficiency, thus supporting their integration into structured medical training programs [7,8]. In addition to virtual reality (VR) simulators, several robotic platforms have significantly contributed to the development of robot-assisted minimally invasive surgery. The da Vinci Surgical System has become the most widely adopted clinical robotic platform, providing enhanced dexterity, three-dimensional visualization, and motion scaling for complex surgical procedures [9]. Other robotic systems, including Hugo RAS, Versius, and Revo-i, have been developed to improve ergonomics, accessibility, and cost-effectiveness while maintaining surgical precision [10,11,12]. Parallel robotic architectures have also attracted considerable attention because of their superior structural stiffness, high positioning accuracy, and reduced moving masses compared with conventional serial manipulators.

Beyond the development of robotic platforms and conventional VR-based training environments, recent research has increasingly focused on Digital Twin (DT) technologies capable of establishing bidirectional connections between virtual and physical robotic systems. Digital Twin architecture has been successfully applied in manufacturing, rehabilitation robotics, industrial automation, medical robotics, and surgical training to enable real-time monitoring, simulation, predictive maintenance, intelligent control, and remote operation. Several bidirectional Digital Twin frameworks capable of synchronizing virtual and physical systems in real time have already been reported in the literature [13,14]. These systems demonstrated the benefits of continuous data exchange between virtual and physical domains, improving monitoring capabilities, operational awareness, training effectiveness, and support for data-driven decision making.

While considerable advances have been made, relatively few studies have combined immersive VR interaction, Digital Twin synchronization, AI-assisted control, physical robotic execution, and independent motion validation within a unified framework specifically designed for surgical robotics. Existing studies typically focus on individual aspects such as VR-based training, Digital Twin monitoring and synchronization, AI-based prediction, or robotic control as separate components. Consequently, there remains a need for integrated platforms capable of simultaneously supporting intuitive user interaction, real-time correspondence between virtual and physical systems, intelligent assistance, and quantitative validation of robot motion. A comparison of representative surgical robotic systems and the proposed framework is presented in Table 1.

To address this gap, the proposed system combines Digital Twin technology, immersive VR interaction, AI-assisted trajectory refinement, physical robot execution, and external optical motion validation within a single architecture developed for a parallel surgical robotic platform. The resulting framework enables seamless bidirectional communication between virtual and physical environments while providing enhanced training capabilities, intelligent decision support, accurate robot control, and objective assessment of motion accuracy.

A broad range of VR- and XR-based systems has already been proposed in the surgical field. Prior work has explored the use of VR, deep learning, and computer vision for localization and positioning of the ATHENA surgical robot [15], as well as VR-based environments for rehabilitation-related applications [16]. Other studies have reported VR simulators for maxillofacial reconstruction [17], systematic analyses of XR in neurosurgical education [18], immersive interactive systems for surgical simulation [19], and open-source platforms integrating VR and haptic feedback for skull-base surgery [20]. Additional validation studies have shown that VR training can support skill development in neurosurgery [21], robot-assisted liver surgery [22], percutaneous renal access rehearsal [23], and laparoscopic training [24]. Taking together, these works confirm the educational value of VR and XR technologies in improving psychomotor training, procedural understanding, and objective skill assessment.

Despite these advances, pancreatic surgery remains one of the most demanding areas of minimally invasive surgery. The deep anatomical location of the pancreas, its proximity to major vascular and neural structures, and the fragility of pancreatic tissue impose strict requirements on instrument stability, motion precision, and coordination [25,26,27,28]. Even minor technical inaccuracies, such as unstable trajectories, imprecise grasping, or unintended tissue contact, may lead to severe intraoperative and postoperative complications. Moreover, trocar-based access further constrains motion and visualization, increasing the cognitive and technical burden placed on the surgeon [29,30]. For these reasons, structured preoperative training is particularly important in robotic pancreatic surgery.

In this context, VR-based training offers an effective way to develop fundamental psychomotor skills that are relevant to minimally invasive pancreatic procedures, including accurate robot positioning, controlled instrument insertion through a trocar, stable grasping, and precise manipulation in confined workspaces. Grasp-and-place tasks are especially relevant because they abstract key intraoperative actions such as tissue handling, retraction, and target-oriented placement while enabling objective assessment through metrics such as execution time, coordination, and motion stability [30,31,32].

Motivated by these clinical and educational needs, this work addresses the development of a Digital Twin–based VR framework for an innovative parallel surgical robot, ATHENA, intended for minimally invasive pancreatic applications. While the physical robotic platform is designed for pancreatic surgery, the virtual training environment is intentionally positioned in an anatomical region located inferior to the pancreas, where sufficient working space is available for controlled introductory manipulation tasks. This design choice reduces the initial cognitive and technical burden on novice users and allows them to focus on essential interaction skills before progressing toward more anatomically constrained and clinically realistic scenarios.

The novelty of the proposed framework does not reside solely in the use of a bidirectional Digital Twin architecture, since such architectures have previously been reported in the literature. Instead, the main contribution of this work consists of the integration of four complementary elements within a unified surgical robotic framework: (i) immersive VR-based interaction allowing intuitive robot manipulation, (ii) real-time synchronization between the Digital Twin and the physical ATHENA surgical robot, (iii) an AI-assisted trajectory refinement module operating within a human-in-the-loop paradigm, and (iv) independent motion validation using an external OptiTrack optical tracking system. The combination of these elements provides a complete framework for surgical training, robot interaction, and performance evaluation.

The main contribution of this work is therefore not limited to the development of a VR simulator, but consists of combining interactive training, real-time robot correspondence, AI-assisted control, and external motion assessment within a single Digital Twin framework for medical robotics. The proposed platform is intended for surgical trainees, surgeons involved in minimally invasive pancreatic procedures, and researchers working on robotic surgical systems. By combining immersive interaction, physical robot execution, and data-driven assistance, the framework aims to support both skill acquisition and the development of next-generation robotic interaction systems for surgical applications, with the potential to reduce execution time, improve accuracy, and enhance patient safety and quality of care.

The rest of the paper is structured as follows. Section 2 presents the ATHENA parallel robot and its kinematic structure. Section 3 describes the proposed system architecture, including the physical robotic platform, software framework, graphical user interfaces, artificial intelligence module, VR environment, and sensing and validation strategy. Section 4 presents experimental results and discussion. Finally, Section 5 summarizes the main conclusions and outlines future research directions.

## 2. Description of the ATHENA Parallel Robot

**Figure 1:**
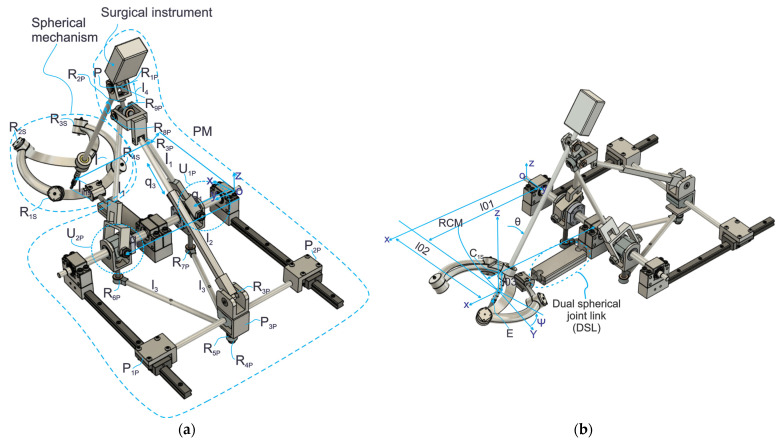
Kinematic structure of the ATHENA parallel robot: (**a**) The geometrical parameters. (**b**) The position of the RCM.

The ATHENA surgical parallel robot (Figure 1) was designed to guide an active laparoscopic instrument and to act as an assistant during complex surgical interventions, such as pancreatectomies [33]. The robot is teleoperated and has a modular architecture composed of two parallel modules. The first module is passive and is based on a spherical parallel mechanism (SPM). Its role is to ensure the Remote Center of Motion (RCM) constraint, so that the surgical instrument consistently passes through the RCM point. This module provides four degrees of freedom (DOF): three for orienting the surgical instrument and one for translation along the instrument axis, allowing instrument insertion and retraction. The translational motion is obtained by replacing one of the revolute joints of the spherical mechanism (SM) with a cylindrical joint, denoted as C_1s_. The SPM is connected to the robot frame through a passive dual-spherical joint link (DSL), which allows the SPM to be positioned above the trocar. The coordinates of the RCM point are determined using three IMU sensors: one mounted on the SPM, one on the DSL element, and one on the robot frame. The second module consists of an active 3-DOF parallel mechanism (PM) composed of three kinematic chains. It is actuated via the active prismatic joints q_1_, q_2_ and q_3_. q_1_ and q_2_ are connected to the links l_1_ through the universal joints U_1p_ and U_2p_. The l_1_ links are connected to each other through the passive revolute joint R_9P_. The active joints q_1_ and q_2_ are connected to the l_3_ links through the revolute joints R_6p_ and R_7p_ and finally R_4p_ and R_5p_. The active prismatic joint q_3_ connects the passive revolute joints R_3P_ and R_8P_, which is connected by the l_2_ links. The surgical instrument is connected to the 3-DOF parallel module through a universal joint composed of two passive revolute joints, R_1P_ and R_2P_, which intersect at point P, corresponding to the center of the revolute joint R_1P_. The parallel robot controls the surgical instrument, which features 4 DOFs: the orientations ψ, and θ, the insertion length l_ins_, or the coordinates of point E (X_E_, Y_E_, Z_E_) along with the orientation angle φ.

The following equation constrains these sets of coordinates:

(1) $$\left\{\begin{array}{l} \psi =\mathrm{atan}2\left(Y_{E},X_{E}\right)\\ \theta =\arccos\left(Z_{E}/\sqrt{{X_{E}}^{2}+{Y_{E}}^{2}+{Z_{E}}^{2}}\right)\\ l_{ins}=\sqrt{{X_{E}}^{2}}+{Y_{E}}^{2}+{Z_{E}}^{2} \end{array}\right.$$

In addition to the geometrical parameters of the robot (l_1_–l_4_) and the length of the surgical instrument (l), the inverse kinematic solution requires the position of the mobile co-ordinate system (oxyz) with respect to the fixed coordinate system (OXYZ), defined by the coordinates (l_01_, l_02_, l_03_). These parameters are subsequently used to determine the active joint coordinates q_1_, q_2_, and q_3_.

Point P is located at the following coordinates:

(2) $$\left\{\begin{array}{l} X_{P}=\left(l-l_{ins}\right)\cdot \cos\left(\psi \right)\sin\left(\theta \right)\\ Y_{P}=\left(l-l_{ins}\right)\cdot \sin\left(\psi \right)\cos\left(\theta \right)\\ Z_{P}=\left(l-l_{ins}\right)\cdot \cos\left(\psi \right)\\ \lambda =\mathrm{atan}\left(Z_{P}-l_{03},X_{P}-l_{01}\right) \end{array}\right.$$

Equation (3) presents the input–output relationships of the ATHENA robot, serving as the basis for deriving the inverse kinematics (active joints q_1_, q_2_, q_3_).

(3) $$\begin{array}{l} f_{1}:l_{02}+(q_{1}+(q_{2}/2))-Y_{P}=0\\ f_{2}:{\left(l_{4}+\sqrt{l_{1}^{2}-{\left(q_{2}-(q_{1}/2)\right)}^{2}}\right)}^{2}-{\left(Z_{P}-l_{03}\right)}^{2}-{\left(X_{P}-l_{01}\right)}^{2}=0\\ f_{3}:{\left(q_{3}+l_{2\min}+l_{5}\right)}^{2}-{\left(X_{P}-l_{01}-l_{4}\cos\left(\lambda \right)+\sqrt{l_{3}^{2}-{\left(q_{2}-(q_{1}/2)\right)}^{2}}\right)}^{2}-{\left(Z_{P}-l_{4}\sin\left(\lambda \right)-l_{03}\right)}^{2}=0 \end{array}$$

so that:

(4) $$\begin{array}{l} q_{1}=Y_{p}-l_{02}-\sqrt{-{\left(X_{p}+l_{01}\right)}^{2}-{\left(Z_{p}+l_{03}\right)}^{2}+l_{1}^{2}-l_{4}^{2}+2\sqrt{{\left(X_{p}l_{4}-l_{01}l_{4}\right)}^{2}+{\left(Z_{p}l_{4}-l_{03}l_{4}\right)}^{2}}}\\ q_{2}=Y_{p}-l_{02}+\sqrt{-{\left(X_{p}+l_{01}\right)}^{2}-{\left(Z_{p}+l_{03}\right)}^{2}+l_{1}^{2}-l_{4}^{2}+2\sqrt{{\left(X_{p}l_{4}-l_{01}l_{4}\right)}^{2}+{\left(Z_{p}l_{4}-l_{03}l_{4}\right)}^{2}}}\\ q_{3}=-l_{2\min}-l_{5}+1/2(-4l_{4}\cos(\lambda )\sqrt{4{l_{3}}^{2}-{\left(q_{1}+q_{2}\right)}^{2}}-8X_{p}l_{4}\cos(\lambda )+8l_{01}l_{4}\cos(\lambda )\\ -8\sin(\lambda )Z_{p}l_{4}+8\sin(\lambda )l_{03}l_{4}+4X_{p}\sqrt{4{l_{3}}^{2}-{\left(q1+q2\right)}^{2}}-4l_{01}\sqrt{4{l_{3}}^{2}-{\left(q_{1}+q_{2}\right)}^{2}}\\ +4{X_{p}}^{2}-8X_{p}l_{01}+4{Z_{p}}^{2}-8Z_{p}l_{03}+4{l_{01}}^{2}+4{l_{03}}^{2}+4{l_{3}}^{2}+4{l_{4}}^{2}-{\left(q1+q2\right)}^{2}{)}^{1/2} \end{array}$$

The ATHENA architecture was selected following a design process focused on the requirements of minimally invasive pancreatic surgery, including workspace characteristics, implementation of the Remote Center of Motion (RCM) constraint, structural stiffness, positioning accuracy, compactness, and patient accessibility.

Compared with conventional serial manipulators, parallel robotic architectures can provide higher structural stiffness, reduced moving masses, improved positioning accuracy, and enhanced dynamic performance. In the ATHENA robot, the passive spherical mechanism ensures preservation of the RCM constraint, while the active parallel module enables accurate positioning and orientation of the surgical instrument within the operative workspace. The selection of the final architecture was therefore driven by the need to achieve an appropriate balance between workspace size, dexterity, mechanical rigidity, positioning precision, and patient accessibility. Such design considerations are commonly employed in the development and evaluation of medical robotic systems, where architectural choices must satisfy both kinematic performance requirements and clinical constraints [34].

Previous studies dedicated to the ATHENA robotic platform investigated different design configurations, workspace characteristics, and optimization strategies, ultimately leading to the architecture adopted in the present work [35]. These investigations demonstrated that the selected configuration provides a favorable compromise between workspace size, kinematic performance, structural rigidity, and accessibility around the patient, making it suitable for minimally invasive pancreatic interventions.

### Dynamic and Control Architecture of the ATHENA Robot

Although the primary focus of this work is the Digital Twin interaction framework, the behavior of the ATHENA robotic platform is also governed by its actuation and control architecture. The robot is actuated through three motorized prismatic joints driven by stepper motors and B&R servo drivers (B&R Industrial Automation GmbH, Eggelsberg, Austria). The control system is coordinated by a programmable logic controller (PLC), which receives motion commands generated from the VR environment through the C# integration layer.

The control structure follows hierarchical architecture composed of three layers:

1. User interaction layer (VR environment);
2. Command generation and AI-assistance layer;
3. Physical robot control layer.

The user-generated trajectories are first computed in Cartesian space and then transformed into joint-space references through the inverse kinematic model presented in Equations (1)–(4). The resulting joint references are transmitted to the X20 PLC (B&R Industrial Automation GmbH, Eggelsberg, Austria), which generates low-level motion commands for the three active actuators.

During operation, the AI module acts as a supervisory trajectory correction layer. Rather than replacing the user commands, the AI module predicts small corrective actions that improve trajectory smoothness and positioning consistency. Therefore, the overall system remains fully human-in-the-loop.

The quality of the generated motion is subsequently evaluated through external OptiTrack measurements. Consequently, the experimental metrics presented in Section 4 represent the combined performance of the user interaction layer, AI-assisted correction layer, and robot control layer.

## 3. Materials and Methods

### 3.1. System Overview

**Figure 2:**
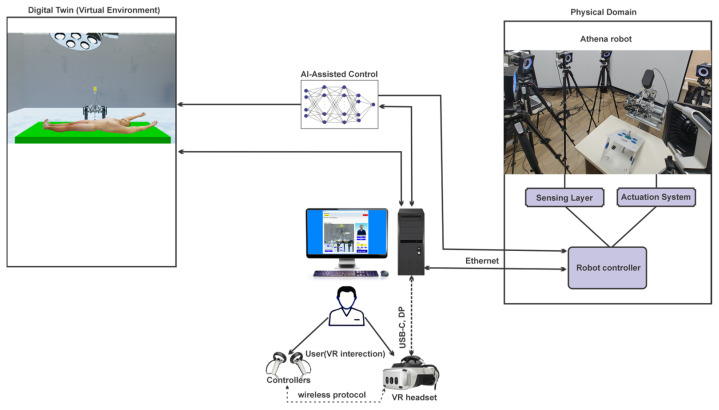
Digital Twin–based system architecture illustrating the bidirectional interaction between the virtual environment and the physical ATHENA robot. Robot motion is controlled through VR interaction, while an external optical tracking system (OptiTrack) is used for independent validation of the robot position.

Figure 2 illustrates the Digital Twin–based architecture of the proposed system, highlighting the bidirectional interaction between the physical ATHENA parallel robot and its virtual counterpart. The framework is structured into two main domains: the physical domain, which includes the robotic platform, and the Digital Twin domain, which is implemented within an immersive virtual reality environment.

In the Digital Twin domain, the virtual environment provides an interactive representation of the robotic system, allowing the user to manipulate the virtual robot using VR controllers. These inputs are translated into motion commands, which are transmitted to the robot controller and executed by the physical system. Thus, the primary control of the robot is achieved through user interaction within the VR environment, ensuring intuitive and immersive operation. In the physical domain, the ATHENA robot executes the commanded motions through its actuation system under the supervision of the robot controller. The resulting robot motion is reflected in the Digital Twin through the continuous exchange of control and execution data, enabling real-time synchronization between the virtual and physical systems.

An external optical tracking system based on OptiTrack (NaturalPoint Inc. 3658 SW Deschutes St, Corvallis, OR, USA) cameras and reflective markers is employed to independently monitor the position and orientation of the physical robot. This system is used for validation and accuracy assessment, providing a reference measurement of robot motion without directly influencing the Digital Twin control loop. An AI-assisted control module is also integrated into the architecture as an optional component, which can be activated to enhance motion quality and provide trajectory assistance when needed. However, the primary control remains user-driven through the VR interface, while the AI module acts as a supplementary layer for performance improvement. Overall, the architecture establishes a bidirectional framework in which the Digital Twin enables intuitive control of the robotic system, the physical robot executes the corresponding motions in real time, and external sensing is used for validation and system accuracy assessment.

**Figure 3:**
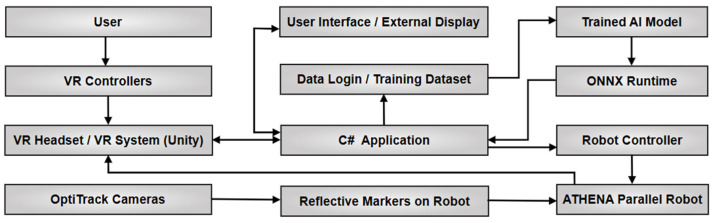
Interconnectivity of the main system components.

Figure 3 illustrates the interconnectivity of the main components of the proposed system, highlighting the data flow between the virtual environment, the control application, and the physical ATHENA robotic platform. The architecture is centered on the interaction between the user, the virtual reality system, and the robot control layer, enabling intuitive manipulation and real-time execution of robot motions. The user interacts with the system through VR controllers and a head-mounted display, which provide an immersive interface for manipulating the virtual representation of the robot within the Unity-based environment. The VR application communicates bidirectionally with the C# application, which acts as a central integration layer for data processing, communication, and system coordination.

The C# application is responsible for transmitting the motion commands generated in the virtual environment to the robot controller, which executes them through the actuation system of the ATHENA parallel robot. At the same time, the application manages the graphical user interface displayed on an external monitor, providing real-time feedback regarding system states, robot variables, and task execution. In addition, the C# application performs data acquisition and logging by storing relevant parameters associated with user interaction and robot motion. These data are used to generate training datasets for the artificial intelligence module. The trained neural network model is exported in ONNX format and subsequently integrated into the system using ONNX Runtime 1.2.1 (Microsoft Corporation, Redmond, WA, USA), allowing efficient inference directly within the C# application.

The AI module operates as an optional component within the control architecture, providing trajectory refinement and motion assistance when activated. However, the primary control of the robotic system remains user-driven through the VR interface, ensuring intuitive and flexible interaction. An external optical tracking system based on OptiTrack cameras and reflective markers is employed to monitor the position and orientation of the physical robot. This subsystem is used exclusively for validation and accuracy assessment and does not directly influence the system control loop.

**Figure 4:**
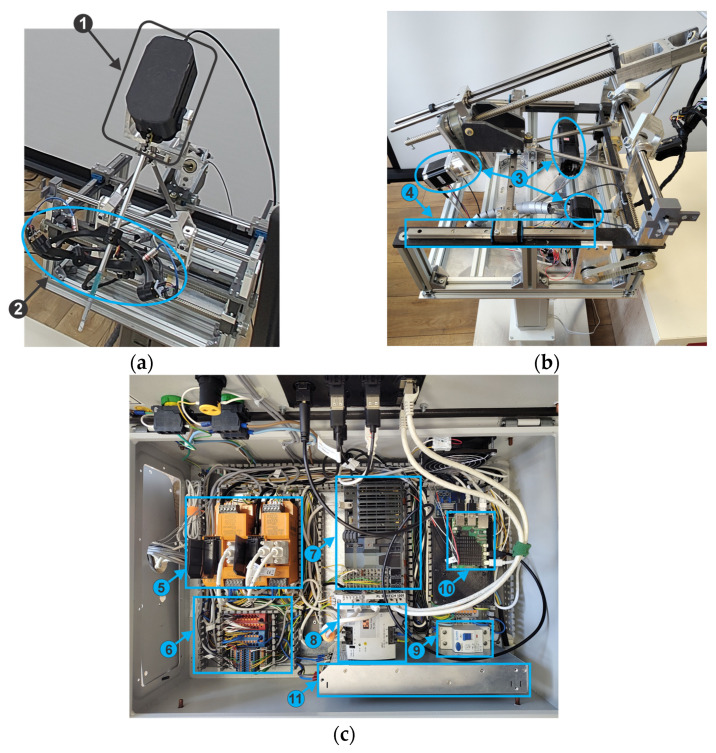
Hardware configuration of the ATHENA robotic platform. The mechanical structure with the active instrument (1) and passive spherical mechanism (2) is shown in (**a**). The actuation subsystem includes stepper motors (3) and linear guides (4), shown in (**b**). The control cabinet (**c**) integrates the B&R motor drivers (5), electrical connectors and terminal blocks (6), PLC (7), 24 V power supply (8), circuit breaker (9), Raspberry Pi 5 embedded computer (10), and the 230 V/24 V power conversion unit (11).

The experimental model of ATHENA is presented in Figure 4. The active instrument, shown in Figure 4a, (1), is mounted on the end-effector and provides controlled insertion and orientation, enabling precise tissue manipulation during surgical procedures. The passive spherical mechanism, shown in Figure 4a, (2), ensures the RCM constraint through a set of passive joints and allows patient-specific adjustment of the insertion point.

**Figure 5:**
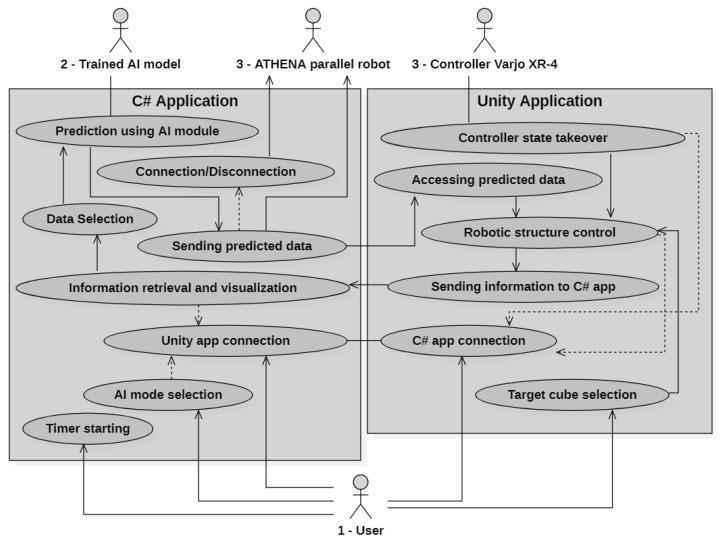
UML use case diagram.

**Figure 6:**
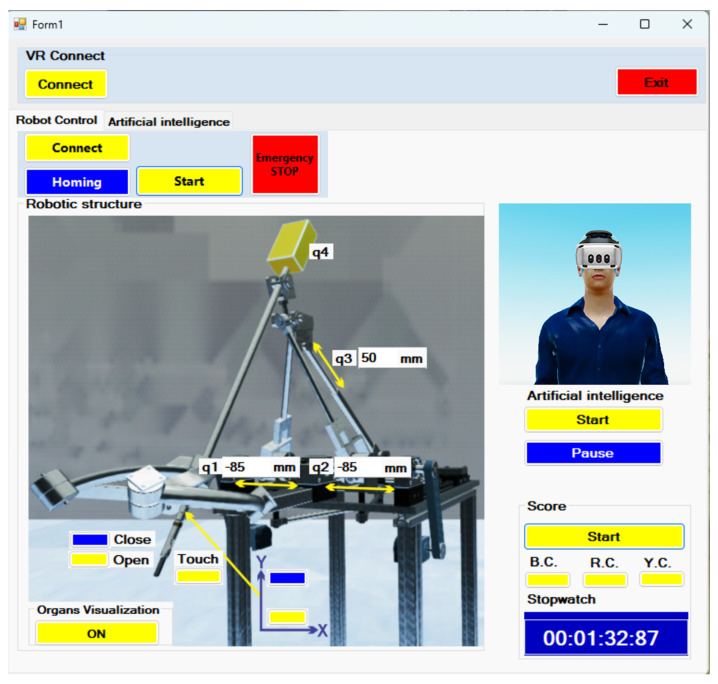
User Interface: Robot control.

Motion generation is achieved using stepper motors, shown in Figure 4b, (3), coupled with a linear guide system, shown in Figure 4b, (4), providing accurate translational actuation of the robot joints. The actuation system is controlled by B&R motor drivers (B&R Industrial Automation GmbH, Eggelsberg, Austria), shown in Figure 4c, (5), which execute the motion commands received from the central control unit. Electrical interconnections are managed through industrial connectors and terminal blocks, shown in Figure 4c, (6), ensuring reliable signal and power distribution.

Beyond the functional interconnectivity illustrated in Figure 3, the real-time operation of the proposed Digital Twin framework is also influenced by the responsiveness of the communication and control chain. System responsiveness depends on several components, including VR controller acquisition, communication between the Unity environment (Unity 3D, version 2022.3.4f1, Unity Technologies, San Francisco, CA, USA) and the C# application, AI inference, Ethernet communication with the robot controller, actuator response, and synchronization between the Digital Twin and the physical robotic system. Although end-to-end latency was not measured as an independent performance metric in the present study, no noticeable delays affecting task execution or user interaction were observed during the experimental sessions. Nevertheless, a comprehensive characterization of end-to-end latency, communication delays, Digital Twin synchronization frequency, controller response time, AI inference contribution, and system update rate will be conducted in future work in order to quantitatively evaluate the real-time performance of the proposed framework.

**Figure 7:**
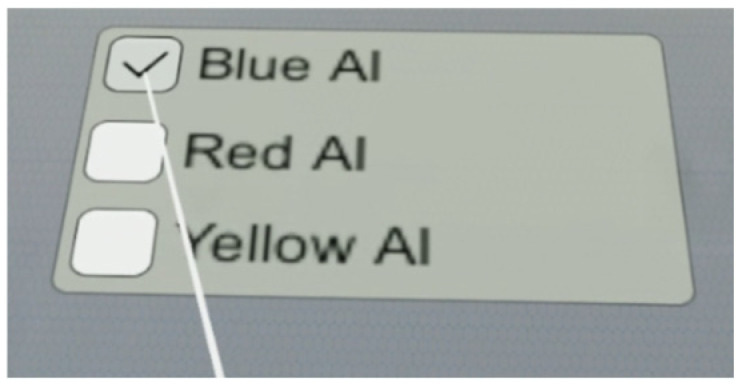
User Interface: VR functionality selection.

**Figure 8:**
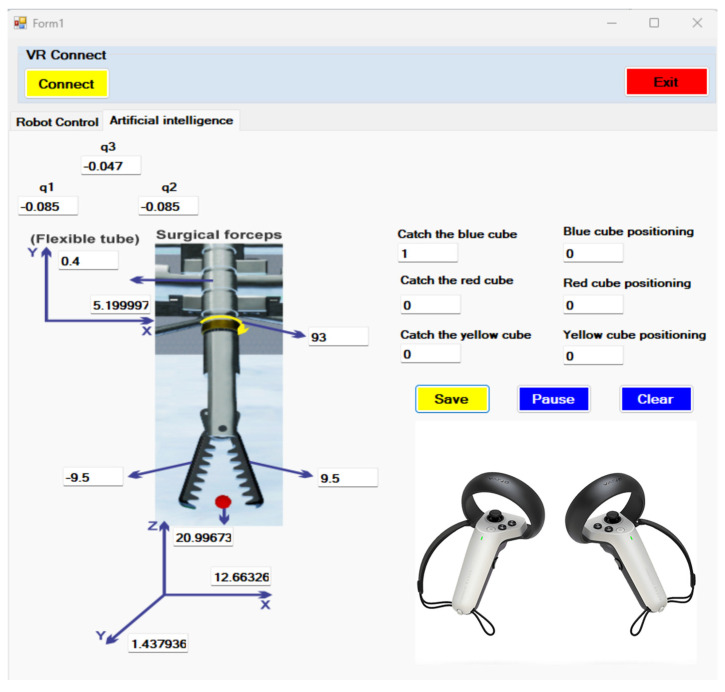
User Interface: Interaction with the AI Module.

**Figure 9:**
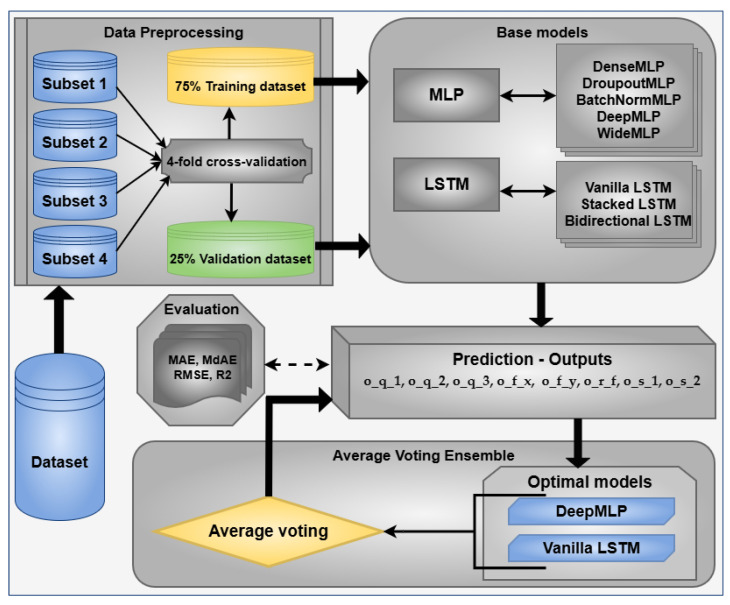
Average voting ensamble architecture.

**Figure 10:**
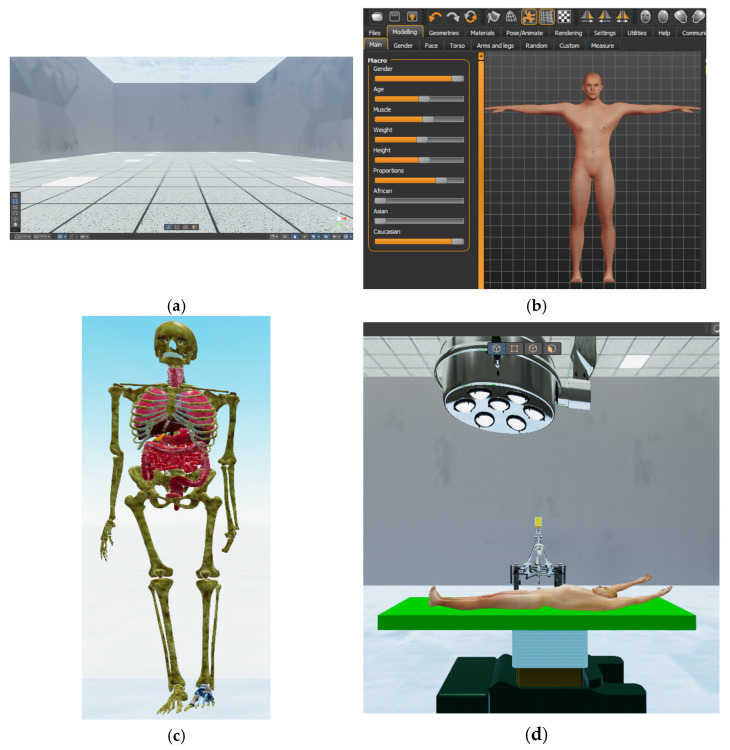
(**a**) Surgical room. (**b**) Virtual patient. (**c**) Skeleton with internal organs. (**d**) Integrating components in the Unity 3D environment.

**Figure 11:**
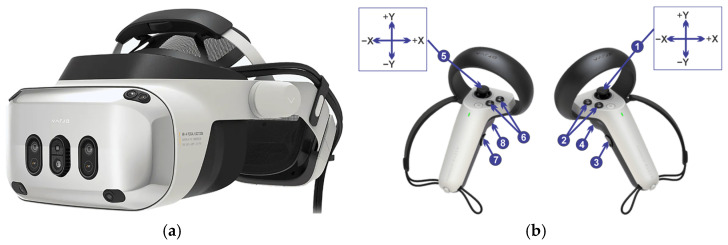
Technologies used in robotic system command and control: (**a**) VR headset; (**b**) controllers.

The control architecture is centered on a PLC with modular extensions, shown in Figure 4c, (7), which coordinates real-time robot operation and communication across all subsystems. A dedicated 24 V power supply, shown in Figure 4c, (8), ensures stable operation of the control components, while an automatic circuit breaker, shown in Figure 4c, (9), provides electrical protection. A Raspberry Pi 5 embedded system, shown in Figure 4c, (10), complements the PLC by handling sensor acquisition, peripheral interfacing, and auxiliary control tasks. Finally, the entire system is powered by a 230 V to 24 V conversion unit, shown in Figure 4c, (11), enabling safe and standardized operation within the experimental ATHENA platform.

### 3.2. Software Application Design

The software architecture of the proposed system is designed to support the Digital Twin–based framework by enabling real-time interaction between the virtual environment, the control application, and the physical ATHENA robotic system. The implementation integrates a Unity-based virtual reality application with a C# control application, forming a unified platform for user interaction, data processing, and robot control. The software layer ensures the bidirectional exchange of information between the virtual and physical domains, allowing the user to interact with the Digital Twin while the corresponding commands are executed by the physical robot. At the same time, the system manages data acquisition, visualization, and optional AI-assisted processing, supporting both real-time operation and data-driven performance enhancement.

The functional architecture of the software system is illustrated through the use case diagram [36] shown in Figure 5. Designed using the Unified Modeling Language [37], the diagram highlights the interaction between the desktop C# application, the Unity-based virtual reality environment, the external control, the ATHENA parallel robot, and intelligence modules. In total, the diagram comprises 14 distinct use cases that define the core functionalities of the system, of which eight are associated with the C# application and six with the Unity VR application. These use cases collectively cover data selection, AI-based prediction, communication between software modules, ATHENA robotic system control, and visualization of information.

Four primary actors are identified: the human user operating the software, the ATHENA parallel robot, the trained artificial intelligence model developed in Python 3.12 (Python Software Foundation, Wilmington, DE, USA), and the Varjo XR-4 controller (Varjo Technologies Oy, Vuorikatu 20, 00100 Helsinki, Finland), which provides input for interaction within the virtual environment. The interactions between actors and the C# [38] software system are represented through nine association relationships, while five dependency relationships illustrate the logical and functional dependencies between individual use cases. The use case diagram therefore reflects the functional integration of user interaction, Digital Twin representation, and intelligent control within a unified framework.

### 3.3. Graphical User Interface

The graphical user interface (GUI) represents the human–robot interaction layer of the proposed Digital Twin–based system and is composed of two complementary components: an immersive virtual reality interface and a desktop-based graphical interface. These interfaces operate together to enable real-time control, monitoring, and data acquisition for the parallel surgical robot, as illustrated in Figure 2 and Figure 3.

The primary control of the robotic system is achieved through the virtual reality interface, where the user interacts with the Digital Twin of the ATHENA robot using VR controllers (Varjo XR-4). Within this immersive environment, the user directly manipulates the virtual robot, generating motion commands that are transmitted in real time to the control application and subsequently executed by the physical system.

In addition to motion control, the VR environment provides interactive virtual panels that allow the user to access system functionalities, including the activation and configuration of the artificial intelligence module. Interaction within the immersive VR environment is achieved using a virtual laser pointer controlled by the handheld VR controllers, enabling intuitive activation of the AI-assisted control modes associated with the robotic manipulation tasks. Through this interaction mechanism, the user can activate real-time trajectory refinement and motion assistance functionalities during robot operation. Once selected, the corresponding AI-assisted module is activated to provide trajectory refinement and motion guidance during robot operation. The VR interface therefore enables:

- direct control of the robotic system through manipulation of the Digital Twin;
- intuitive interaction with virtual control elements;
- real-time visualization of the robotic task and workspace.

Through this approach, the VR interface acts as the primary control interface, directly influencing the behavior of the physical robotic system.

The robot control interface, implemented within the desktop-based GUI, provides supervision and monitoring functionalities for the commands generated in the VR environment. This interface enables the user to manage system operation and verify the execution of robotic tasks, while maintaining synchronization between the virtual and physical systems.

The interface includes functionalities related to system initialization, command supervision, and task evaluation, as illustrated in Figure 6. In addition to monitoring capabilities, the interface incorporates a set of control commands that enable direct interaction with the physical ATHENA robot, ensuring proper system operation and safety. These control commands include:

- Connection control: A dedicated command allows the establishment of communication between the control application and the physical robot, enabling real-time synchronization between the VR environment and the robotic system.
- Homing procedure: The interface provides functionality for initializing the robot by setting it to a predefined reference configuration, ensuring consistent starting conditions for operation.
- System activation: A control command is used to start the execution of the robotic system, allowing the physical robot to follow the motion generated within the virtual environment.
- Emergency stop mechanism: A safety-critical function is implemented to immediately halt the operation of the physical robot in case of unexpected behavior or emergency conditions.

These control functionalities ensure reliable initialization, execution, and safe operation of the robotic system, while maintaining coherence between the Digital Twin and the physical robot. The interface also provides real-time visualization of system states and task execution parameters, including actuation variables associated with the robotic system, spatial positioning of the surgical instrument, operational state of the end-effector (e.g., open/close state), interaction feedback, such as contact detection during object manipulation and task execution status, including performance evaluation metrics.

The VR functionality selection interface, which allows the user to activate and configure the AI-assisted control modes, is shown in Figure 7.

The artificial intelligence interface supports both data acquisition and interaction with the AI-assisted control module. Through this interface, the data generated by the VR controllers (Varjo XR-4), used to operate the robotic system, are displayed and recorded for further processing. The recorded data include control inputs and system variables associated with robot operation, as illustrated in Figure 8, such as joint actuation variables (q_1_, q_2_, q_3_) which defines the motion of the parallel robotic structure, spatial positioning parameters (X, Y) of the flexible surgical instrument, rotational control of the surgical instrument, end-effector state, including opening and closing of the surgical forceps or the cartesian coordinates (X, Y, Z) of the end-effector reference point.

The trained models are integrated into the system using ONNX Runtime, as described in Section 3.1. When activated, the AI module uses these models to provide trajectory refinement and motion assistance. However, the AI component operates as an optional support layer, while the primary control remains user-driven through the VR interface.

### 3.4. Artificial Intelligence Methods Used in Parallel Robot Positioning Estimation

In order to correct the trajectory, stabilize motion, and optimize the process of grasping and positioning the cubes, the proposed system architecture integrates artificial intelligence methods capable of providing real-time assistance to the user.

The dataset used by the AI models is numerical and comprises 17 input variables and 8 output variables. The signals denoted (q_1), (q_2), and (q_3), which represent the kinematic variables of the parallel robot motors, are responsible for positioning the mobile platform in both the horizontal and vertical planes. They describe the internal kinematic state of the parallel robot and are used both for manual control and as input variables for the neural networks.

The signals associated with the flexible tube, denoted (f_x) and (f_y), enable lateral and transverse positioning of the surgical forceps. The signal (r_f) defines the orientation of the surgical instrument required for gripping and manipulation. The variables (s_1) and (s_2), representing the opening/closing angles of the first and second branches of the surgical forceps, respectively, jointly define the gripping state of the instrument. The variables (t_x), (t_y), and (t_z), representing the coordinates of the center point of the surgical forceps along the x-, y-, and z-axes, respectively, describe the spatial position of the active interaction point with the virtual objects.

The target output variables were defined as assistive corrective commands intended to improve manipulation accuracy during task execution. Their values were derived from successful task trajectories recorded during operator interaction and represent the desired control adjustments required to achieve stable grasping and accurate object placement.

Specifically, the variables (o_q_1), (o_q_2), and (o_q_3) provide corrective actions for the active joints of the parallel mechanism, (o_f_x) and (o_f_y) provide corrective positioning commands for the flexible instrument, (o_r_f) represents rotational correction of the forceps, while (o_s_1) and (o_s_2) correspond to optimized forceps opening and closing commands. Together, these outputs constitute the minimum control set required for trajectory refinement and AI-assisted manipulation.

The logical variables associated with the cubes include (b_v), (r_v), and (y_v), which indicate the gripping state of the blue, red, and yellow cubes, respectively, and (b_c), (r_c), and (y_c), which indicate whether each cube has been correctly positioned in the target area. The first three logical variables take the value 1 when the corresponding cube is grasped and 0 otherwise, while the last three variables take the value 1 when the corresponding cube is correctly positioned and 0 otherwise. The structure and meaning of the 17 input variables are presented in Table 2.

The 8 output variables, denoted (o_q_1), (o_q_2), (o_q_3), (o_f_x), (o_f_y), (o_r_f), (o_s_1), and (o_s_2), represent assistive commands intended to support the user during the grasping and positioning of the three cubes. These commands are generated based on the patterns learned during training and include corrections for the parallel mechanism, the flexible tube, and the surgical instrument. Therefore, the output variables enable real-time trajectory correction, spatial position adjustment, and optimization of instrument orientation and gripping.

Based on the structure of the numerical dataset and the nature of the multivariate regression problem, five Multilayer Perceptron (MLP) architectures and three Long Short-Term Memory (LSTM) architectures were selected for analysis and comparative evaluation. These models were chosen because they represent different levels of architectural complexity and generalization capability.

#### 3.4.1. Dataset Acquisition and Validation

The dataset used for neural network training was acquired during repeated executions of a standardized grasp-and-place task performed within the Digital Twin environment. During each execution, the operator manipulated the ATHENA virtual robot using the Varjo XR-4 controllers while the system continuously recorded the corresponding robot states, instrument variables, spatial coordinates, and object interaction states. The acquisition process generated synchronized records containing the 17 input variables described in Table 2. A total of 2000 valid samples were collected from multiple task executions. Prior to training, the dataset was subjected to a validation procedure consisting of signal synchronization verification, numerical range inspection, missing-value detection, duplicate sample removal, and logical consistency checks for the cube interaction variables. Records containing incomplete information or inconsistent state transitions were discarded. Following validation, all variables were normalized to the interval [0, 1] to ensure numerical stability during neural network training. The resulting dataset was subsequently divided into training and validation subsets and evaluated using 4-fold cross-validation.

#### 3.4.2. Machine Learning Implemented Methods

Dense Multilayer Perceptron (DenseMLP) is a fully connected feedforward neural network used as a benchmark model for evaluating baseline performance in multivariate regression problems [39]. Dropout Multilayer Perceptron (DropoutMLP) extends the DenseMLP architecture by introducing dropout regularization, which aims to reduce overfitting and improve generalization capability [40]. Batch Normalization Multilayer Perceptron (BatchNormMLP) integrates batch normalization between dense layers, helping to stabilize the training process and accelerate model convergence [41]. Deep Multilayer Perceptron (DeepMLP) uses an increased number of hidden layers to capture complex nonlinear relationships between input and output variables [42]. Wide Multilayer Perceptron (WideMLP) is characterized by a larger number of neurons per layer, increasing the representation capacity of the model through width rather than depth [43]. Long Short-Term Memory (LSTM) networks are designed to selectively retain or discard information in temporal sequences, making them suitable for modeling long-term dependencies in sequential data [44]. The standard Long Short-Term Memory architecture, referred to here as Vanilla LSTM, consists of a single LSTM layer and is used to model basic temporal dependencies within the data sequence [45]. Stacked Long Short-Term Memory (Stacked LSTM) refers to an architecture composed of multiple LSTM layers arranged sequentially, allowing the model to learn hierarchical representations and more complex temporal relationships [46]. Bidirectional Long Short-Term Memory (Bidirectional LSTM) processes information in both forward and backward directions, enabling the model to capture both past and future context within the sequence [47].

The eight neural network architectures were optimized by adjusting architecture-specific hyperparameters, whose selected optimal values are summarized in Table 3.

The optimization process was conducted using the Optuna framework [48], which enabled automated exploration of the predefined search spaces. The final configuration of each model was selected based on the minimization of the validation error, ensuring a balance between predictive accuracy and generalization capability. The resulting hyperparameter configurations reflect the structural particularities of each architecture, with deeper or wider MLP variants requiring different learning rates, numbers of layers, and training epochs compared with the LSTM-based models.

Since the analyzed problem is a multivariate regression problem, the eight neural networks and the hybrid ensemble were evaluated using four metrics: Mean Absolute Error (MAE), Median Absolute Error (MdAE), Root Mean Square Error (RMSE), and the coefficient of determination (R^2^) [49]. These metrics provide a comprehensive assessment of predictive performa1nce by quantifying average absolute deviations, robustness to outliers, penalization of larger errors, and the proportion of variance explained by the model.

The dataset was partitioned into 75% for training and 25% for validation, corresponding to 1500 and 500 samples, respectively, out of the total 2000 records available for each variable. In addition, the evaluation was conducted within a 4-fold cross-validation framework to improve robustness and reduce the variability associated with a single data split. This procedure enhances the reliability of the reported performance indicators and supports the validity of the comparative analysis.

The analysis of the obtained metrics shows that the DeepMLP and Vanilla LSTM models achieved the best individual performances. The larger differences between training and validation metrics observed for the MLP-based networks indicate a higher sensitivity to overfitting, whereas the LSTM-based architectures showed more stable behavior and better generalization capability.

The values obtained for the selected metrics during both the training and validation phases are summarized in Table 4. Based on these observations, an Average Voting Ensemble [50], illustrated in Figure 9, was proposed by combining the predictions of the DeepMLP and Vanilla LSTM networks.

The initial dataset was subjected to preprocessing and 4-fold cross-validation, followed by division into training and validation subsets. The final prediction was obtained by combining the outputs of the two models using an average voting strategy, exploiting the complementarity between the nonlinear approximation capability of the DeepMLP model and the temporal stability of the Vanilla LSTM architecture. The contribution of the Vanilla LSTM model was particularly important in reducing the discrepancy between training and validation performance, thus providing a regularization effect within the ensemble.

Several measures were adopted to reduce the risk of overfitting. First, the dataset was divided into independent training (75%) and validation (25%) subsets. Second, a 4-fold cross-validation procedure was applied to reduce the dependency of the results on a single train–validation split. Third, regularization-oriented architectures, including DropoutMLP and BatchNormMLP, were evaluated during model selection. Finally, the proposed Average Voting Ensemble combined DeepMLP and Vanilla LSTM models, providing improved validation stability and reducing the discrepancy between training and validation performance.

The validation results reported in Table 4 show only a small difference between training and validation metrics for the final ensemble model (R^2^ = 0.99693 for training and R^2^ = 0.98801 for validation), indicating good generalization within the investigated dataset and no evidence of significant overfitting. Nevertheless, additional validation involving larger participant groups and more complex surgical scenarios will be considered in future studies to further assess model robustness.

The results obtained on the validation dataset confirm the superior predictive capability of the proposed ensemble, which records the lowest validation error values (MAE = 0.02284, RMSE = 0.03159) and the highest coefficient of determination (R^2^ = 0.98801), outperforming all individual neural network models.

These results demonstrate the effectiveness of combining complementary architectures in order to enhance overall predictive accuracy. Moreover, the reduced discrepancy between the training and validation metrics indicates stable model behavior and the absence of significant overfitting. The voting ensemble preserves the representational strength of the DeepMLP model while benefiting from the improved generalization stability provided by the Vanilla LSTM architecture. Therefore, the integration of the proposed voting ensemble based on DeepMLP and Vanilla LSTM neural networks demonstrates high potential for precise and robust assistance of the positioning and manipulation process within parallel robotic systems.

### 3.5. Development of the Virtual Reality Application

The VR application represents the core interaction environment of the proposed Digital Twin–based system, enabling intuitive control, real-time visualization, and task execution for the ATHENA parallel surgical robot. The virtual environment was developed in Unity using the ProBuilder package [51,52], which enabled rapid construction and refinement of the workspace without the need for external modeling software. To provide a realistic surgical context, a virtual patient model was generated using MakeHuman [53] and extended with a skeletal structure and internal organs, as illustrated in Figure 10.

The robotic structure and surgical instrument constitute the main components of the Digital Twin. These elements were designed in Siemens NX, where the robot architecture, degrees of freedom, motion constraints, and grasper mechanism were modeled and validated. The final assembly was then exported to Unity, ensuring consistency between the virtual and physical systems while maintaining real-time interactivity. Figure 10d presents the main components integrated into the VR environment.

Within the VR application, the user interacts with the Digital Twin using Varjo XR-4 controllers. The controller motions are mapped to the corresponding movements of the virtual robot, generating commands that are transmitted to the control application and subsequently executed by the physical system. This establishes a real-time interaction loop between user actions, virtual robot motion, and physical robot execution, as shown in Figure 2 and Figure 3.

The application supports manipulation tasks, such as grasping and positioning objects with the surgical instrument, allowing both user interaction and robotic performance to be evaluated. During task execution, the user controls the position, orientation, and grasper state of the instrument while receiving continuous visual feedback. Interactive menus and virtual control panels also allow the configuration of system parameters and the activation of auxiliary functions, including the AI-assisted control module.

In addition to control and visualization, the VR system acts as a data acquisition source by recording user-generated inputs that are subsequently used for training the artificial intelligence models. Overall, the VR application provides a unified platform for immersive interaction, real-time robot control, simulation, and data-driven optimization within the proposed Digital Twin framework.

### 3.6. Using the VR Environment

The use of surgical robots and specialized simulators has gained significant attention due to their ability to improve the accuracy of interventions, reduce intra-operative risks and support the effective training of medical staff. Through the simulator, the user can train in the use of the surgical robot by practicing the maneuvering of a surgical forceps with which various objects, such as three cubes, are manipulated. To control the surgical robot within the VR application, the user uses two controllers (Figure 11b). These allow intuitive and precise interaction with the robotic system, facilitating the positioning and execution of different simulated surgical operations.

**Figure 12:**
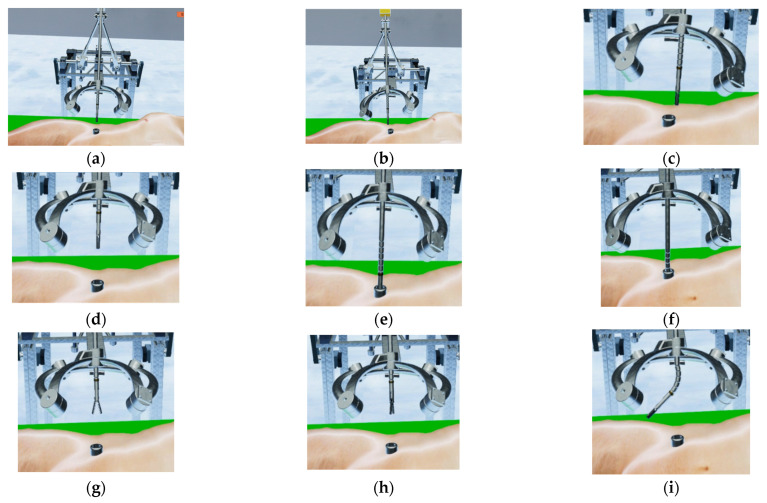

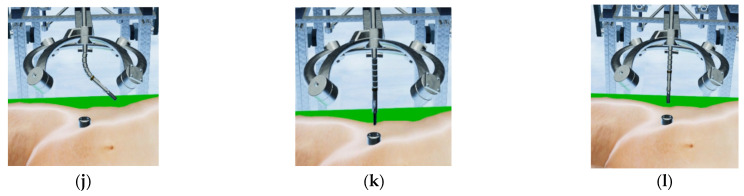
Experimental task performed in the Digital Twin environment. Legend: (**a**) start position; (**b**) position 1 (−X translation); (**c**) position 2 (+X translation); (**d**) position 3 (+Y translation); (**e**) position 4 (−Y translation); (**f**) insertion and withdrawal of the surgical instrument; (**g**) opening and closing of the surgical forceps; (**h**) 360° rotation of the surgical forceps; (**i**) position 1 of the surgical forceps (−X bending); (**j**) position 2 of the surgical forceps (+X bending); (**k**) position 3 of the surgical forceps (−Y bending); (**l**) position 4 of the surgical forceps (+Y bending).

Using the controllers, the user can position the robot to perform the following actions as follows:

- Start position (Figure 12a)—the initial position of the robotic system, from which the simulation starts;
- Position 1 (Figure 12b)—obtained by sliding the slider on the controller along the −*X* axis (Figure 11b, (1));
- Position 2 (Figure 12c)—obtained by sliding the slider on the controller on the +*X* axis (Figure 11b, (1));
- Position 3 (Figure 12d)—obtained by sliding the slider on the controller on the +*Y* axis (Figure 11b, (1));
- Position 4 (Figure 12e)—obtained by sliding the slider on the controller on the −*Y* axis (Figure 11b, (1));
- Insertion and withdrawal of the surgical instrument (Figure 12f)—achieved by pressing two dedicated buttons (Figure 11b, (2));
- Opening and closing of the surgical forceps (Figure 12g)—opening is activated by pressing the button (Figure 11b, (7)) and closing by pressing the button (Figure 11b, (8));
- Rotating the surgical forceps 360° (Figure 12h)—accomplished by alternately pressing two buttons (Figure 11b, (6));
- Position 1 of the surgical forceps (Figure 12i)—achieved by directional bending of the flexible tube on the −*X*-axis (Figure 11b, (5));
- Position 2 of the surgical forceps (Figure 12j)—obtained by bending the flexible tube in the +*X* axis (Figure 11b, (5));
- Position 3 of the surgical forceps (Figure 12k)—obtained by bending the flexible tube in the −*Y* axis (Figure 11b, (5));
- Position 4 of the surgical forceps (Figure 12l)—obtained by bending the flexible tube on the +*Y* axis (Figure 11b, (5)).

The cube manipulation task was intentionally designed as a simplified benchmark scenario rather than as a direct representation of pancreatic surgery. The primary objective of this task was to evaluate fundamental psychomotor skills required in minimally invasive robotic procedures, including precise instrument positioning, controlled grasping, hand–eye coordination, trajectory planning, and target-oriented object manipulation. Furthermore, the task provides a controlled and repeatable environment for assessing user interaction, AI-assisted guidance, and Digital Twin synchronization.

Although the task captures several essential components of robotic manipulation, it does not reproduce the full complexity of surgical procedures, including tissue deformation, force interaction, anatomical variability, bleeding, restricted visibility, or other clinically relevant factors. Consequently, the presented experimental scenario should be regarded as an initial validation of the proposed framework rather than a complete surgical simulation. Future work will focus on extending the Digital Twin environment toward anatomically realistic surgical scenarios involving deformable tissue models, procedure-specific tasks, and clinically relevant training protocols.

### 3.7. Experimental Protocol

Six participants with previous experience in VR interaction were recruited for the study. Each participant performed the same object manipulation task under two operating conditions:

- Conventional VR control without AI assistance;
- VR control with AI-assisted trajectory refinement.

The task consisted of grasping three colored cubes and placing them in predefined target locations. For each trial, the completion time, robot trajectory, and system state variables were recorded. The sequence of operations was identical for all participants. Before the experiments, a short familiarization session was conducted to minimize learning effects. During the experiments, the participants were instructed to complete the task as quickly and accurately as possible.

### 3.8. Motion Validation Methodology

The physical robot motion was independently measured using an OptiTrack optical tracking system equipped with multiple infrared cameras and reflective markers attached to the robot structure. A calibration procedure was performed to align the coordinate frames of the Unity virtual environment, the robot controller, and the OptiTrack measurement system.

For each sampled point, the Euclidean distance between the reference position generated by the Digital Twin and the corresponding position measured by OptiTrack was computed. The resulting errors were used to calculate mean position error, root mean square error (RMSE) and maximum error.

These metrics were used to quantify the correspondence between the Digital Twin and the physical robotic system.

## 4. Results and Discussion

### 4.1. Validation of Performance Evolution in the Virtual Environment

**Figure 13:**
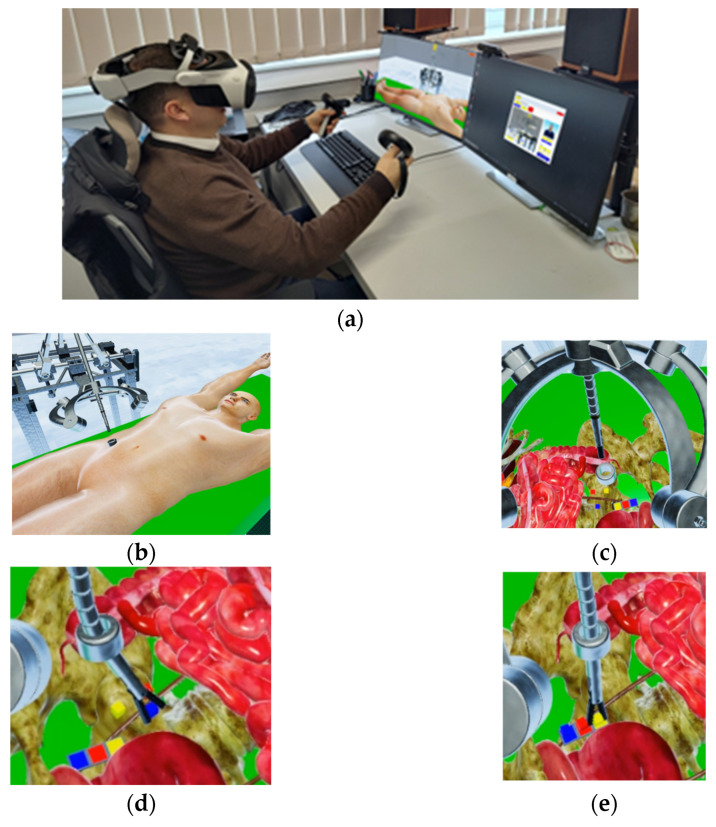

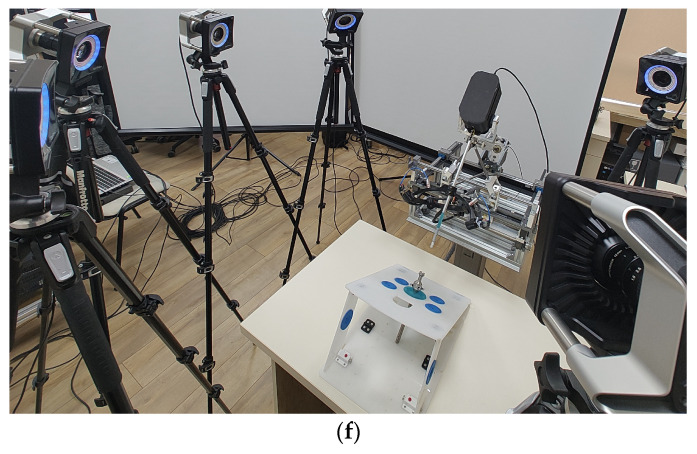
Experimental VR training and physical validation: (**a**) immersive VR interaction; (**b**) robot positioning for instrument insertion through the trocar; (**c**) transparency-based visualization of internal anatomy; (**d**) cube grasping with the surgical forceps; (**e**) cube placement into the target zones; (**f**) external motion validation using the OptiTrack system.

The integration of VR technologies into robotic surgical training offers significant benefits, including improved technical performance, reduced task completion times, and safe skill acquisition in a controlled simulation environment [54]. In the developed simulator, the user performs a structured training task that reproduces essential steps of robot-assisted minimally invasive procedures. First, the ATHENA robot must be positioned so that the surgical instrument can be inserted through a trocar placed on the surface of the virtual patient, as shown in Figure 13a,b.

To improve spatial awareness, a transparency function can be activated using the VR controller, allowing visualization of the internal anatomical structures, as illustrated in Figure 13c. After insertion, the user performs a grasp-and-place task by sequentially manipulating three colored cubes with the surgical forceps and positioning them in their corresponding target zones, as shown in Figure 13d,e. The task evaluates coordination, precision, and motion stability, while execution time is recorded automatically from correct robot positioning until all cubes are successfully placed.

The framework also extends VR-based interaction to the physical ATHENA platform, which acts as the real counterpart of the Digital Twin. Motion commands generated in the VR environment are transmitted to the control system and executed by the physical robot, enabling synchronized operation between the virtual and physical domains. An optional neural-network-based AI module can be activated to provide assistive trajectory guidance during instrument insertion and object manipulation. This module does not replace user control, but supports motion stability, positioning accuracy, and execution efficiency.

For external validation, an OptiTrack motion capture system was used to measure the physical motion of the ATHENA robot, as shown in Figure 13f. Reflective markers were attached to relevant elements of the robotic structure and to the trocar region, allowing the position and orientation of the robot to be reconstructed during task execution. The motion capture system was not included in the real-time control loop, but was used only as an independent reference for assessing the correspondence between the VR-generated commands and the actual robot motion.

To quantify this correspondence, the trajectory recorded in the Unity-based Digital Twin was compared with the trajectory measured by the OptiTrack system. Before error computation, the datasets were synchronized in time and expressed in a common coordinate frame using the known trocar position and the initial calibrated pose of the instrument. The position error was then calculated at each sample as the Euclidean distance between the commanded point in the VR environment and the corresponding measured point on the physical robot.

To quantitatively assess the correspondence between the Digital Twin and the physical robotic system, the trajectory generated in the Unity virtual environment was compared with the trajectory independently measured by the OptiTrack motion capture system. Prior to the analysis, both datasets were synchronized in time and transformed into a common coordinate frame using the calibrated trocar position and the initial robot pose. The position error was calculated for each sample as the Euclidean distance between the virtual trajectory point and the corresponding OptiTrack measurement:

(5) $$e_{i}=\sqrt{{\left({x_{i}}^{VR}-{x_{i}}^{Opt}\right)}^{2}+{\left({y_{i}}^{VR}-{y_{i}}^{Opt}\right)}^{2}+{\left({z_{i}}^{VR}-{z_{i}}^{Opt}\right)}^{2}}$$

**Figure 14:**
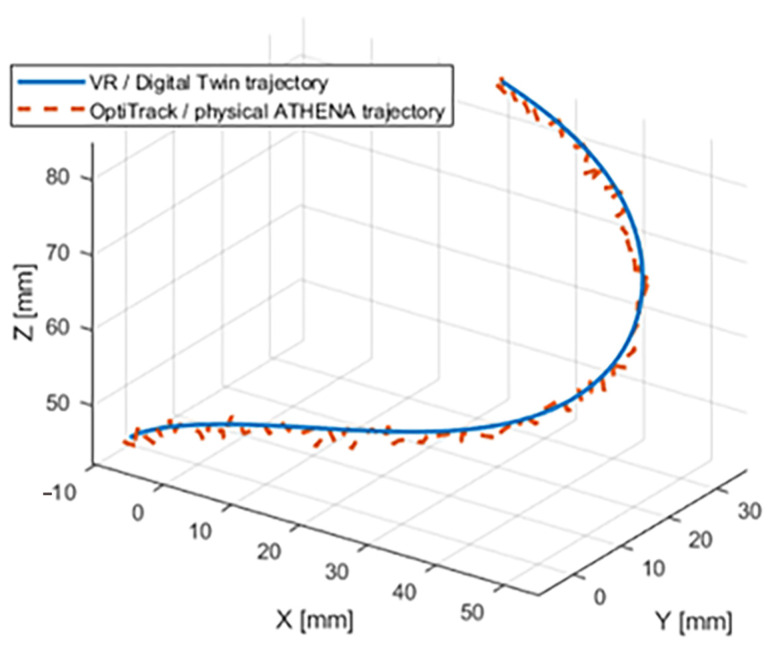
Comparison between the trajectory generated in the VR-based Digital Twin environment and the trajectory executed by the physical ATHENA robot, measured using the OptiTrack motion capture system. The difference between the two trajectories was used to compute the position tracking error.

The statistical indicators presented in Figure 14 were subsequently computed from the complete error sequence. The mean error was obtained as the arithmetic average of all position errors, while RMSE was computed according to:

(6) $$RMSE=\frac{1}{N}\sum_{i=1}^{N}e_{i}^{2}$$

The maximum error corresponds to the largest recorded deviation between the virtual and physical trajectories.

For the representative trajectory shown in Figure 14, the physical robot followed the VR-generated motion with small deviations. The mean position error was 2.42 mm, the root mean square error was 2.56 mm, and the maximum error was 4.68 mm. The largest deviations occurred mainly during changes in direction, where small delays between the virtual command and the physical response became more visible. These errors may be attributed to communication latency, mechanical compliance, calibration uncertainty between the OptiTrack and Digital Twin coordinate frames, and the finite resolution of the actuation and tracking systems. Nevertheless, the small deviations support the feasibility of using the VR environment as an intuitive control interface for the physical ATHENA robot and strengthen the validity of the proposed Digital Twin architecture.

The mean tracking error of 2.42 mm further indicates a close correspondence between the trajectory generated within the Digital Twin environment and the motion executed by the physical ATHENA robot under the investigated experimental conditions. Nevertheless, this value should not be interpreted as a definitive indicator of clinical readiness. Acceptable positioning errors in minimally invasive surgery depend on the specific procedure, anatomical structures involved, instrument dimensions, and safety margins.

Therefore, the reported accuracy should primarily be regarded as evidence of effective synchronization between the virtual and physical components of the proposed system. The obtained results confirm that the Digital Twin framework is capable of maintaining a consistent correspondence between the VR-generated commands and the physical robot motion during task execution. However, additional studies involving anatomically realistic surgical phantoms, tissue interaction, force feedback, and clinically representative trajectories will be required before clinical deployment can be considered.

### 4.2. Experimental Validation

To evaluate the influence of the artificial intelligence module on task execution, a preliminary experimental study was conducted using the VR-based Digital Twin framework described in Section 4.1. The objective of this evaluation was to assess the effect of AI-assisted control on the efficiency of user interaction during robotic manipulation tasks. The pick-and-place task was intentionally selected as an introductory manipulation scenario. Although simpler than clinically representative laparoscopic procedures such as suturing, needle passing, or tissue handling, this task allows objective evaluation of robot positioning accuracy, grasping capability, trajectory stability, and user interaction performance. Future studies will extend the validation protocol toward more realistic minimally invasive surgical training tasks.

A preliminary usability study was conducted involving six participants with engineering backgrounds and prior experience in robotics and virtual reality systems. The objective of the study was not clinical validation but rather evaluation of the Digital Twin interaction framework, user control performance, and system feasibility. Consequently, the results should be interpreted as a preliminary engineering assessment rather than a surgical validation study. All participants provided informed consent prior to taking part in the study.

Each participant was required to perform the same standardized grasp-and-place task described in Section 4.1 under two operating conditions: manual control through the VR interface without AI assistance, and VR-based control with the AI-assisted module activated. In both cases, the task consisted of positioning the robotic system, inserting the surgical instrument through the trocar, and sequentially grasping and placing three colored objects in their corresponding target areas. The performance metric used for comparison was the total task completion time, which was measured automatically using the integrated timing system shown in Figure 6.

To determine whether the reduction in execution time achieved through AI assistance was statistically significant, a paired *t*-test was performed using the completion times recorded from the six participants under both experimental conditions (AI disabled and AI enabled). Since each participant performed the task in both conditions, a paired analysis was considered appropriate.

The average completion time decreased from 150.69 ± 24.22 s in the manual condition to 92.39 ± 18.82 s when AI assistance was activated, corresponding to a mean reduction of 58.30 s (38.69%). The paired *t*-test revealed a statistically significant improvement in task performance (t(5) = 18.52, *p* = 8.5 × 10^−6^). Furthermore, the calculated effect size was extremely large (Cohen’s d = 7.56), indicating a substantial practical impact of the AI-assisted guidance. These results demonstrate that the proposed AI-assisted Digital Twin framework significantly improves task execution efficiency while maintaining user control of the robotic system.

**Figure 15:**
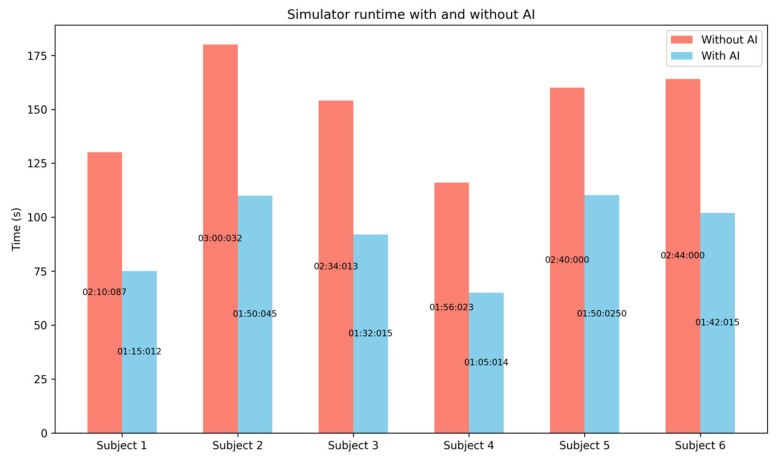
User performance results while using the simulator.

The experimental results are illustrated in Figure 15, which compares the execution times obtained by each participant under the two operating conditions. For all participants, the AI-assisted mode resulted in a shorter task completion time compared with manual control without assistance. This improvement may be attributed to the ability of the AI module to provide smoother and more stable control signals, particularly during critical phases such as instrument insertion and object placement. By refining user-generated commands, the AI-assisted mode reduces abrupt motion variations and facilitates more precise manipulation of the surgical instrument.

It is important to emphasize that the AI module operates as an optional support layer and does not replace user control. The user remains responsible for generating motion commands through the VR interface, while the AI module contributes by improving the quality and stability of these commands. Although the number of participants included in this preliminary engineering study is limited and does not allow clinical conclusions to be drawn, the consistent reduction in task completion time observed across all subjects suggests that AI-assisted interaction can improve task execution efficiency within the proposed Digital Twin framework.

### 4.3. Discussion

The results obtained in this study demonstrate the feasibility of integrating immersive VR, real-time robot interaction, and AI assistance within a unified Digital Twin–based framework for surgical robotic systems. Unlike conventional VR simulators, the proposed approach establishes a direct connection between the virtual environment and the physical ATHENA robot, enabling both training and real-time robotic interaction. The VR environment acts as the primary control interface, allowing users to manipulate the robotic system intuitively through its Digital Twin. This interaction approach enhances spatial perception and user engagement, which are important aspects of surgical training and skill acquisition.

The experimental results show that the proposed system supports the consistent execution of manipulation tasks in a controlled and safe environment. The selected pick-and-place task was intended as a preliminary manipulation benchmark rather than a clinically representative surgical procedure. Its purpose was to evaluate fundamental interaction, positioning, and grasping capabilities before progressing toward more complex laparoscopic training scenarios. The use of task completion time as a performance metric enables an objective evaluation of user interaction efficiency. The AI-assisted module further contributes to improved performance by providing trajectory guidance and motion refinement, as reflected by the reduction in execution time observed across participants. However, the AI component remains an assistive layer and does not replace user control, thereby preserving the human-in-the-loop principle required in medical robotics.

Another important aspect of the proposed approach is the integration of the physical ATHENA robot as the execution layer of the Digital Twin. Motion commands generated in the VR environment are transmitted to the physical robot, enabling synchronized operation between the virtual and real systems. The OptiTrack-based tracking system provides an independent validation layer by measuring the robot position and orientation without influencing the control loop. This allows objective assessment of the correspondence between commanded and executed motions.

Several limitations should be acknowledged. First, the experimental validation was conducted using a limited number of participants, which restricts the statistical significance and generalizability of the results. Furthermore, the participants involved in this preliminary study had engineering backgrounds and experience with robotics and virtual reality systems, rather than clinical surgical expertise. Future studies should therefore include larger cohorts involving surgical residents and experienced surgeons.

Second, the selected pick-and-place task represents a simplified manipulation scenario. While suitable for evaluating positioning accuracy, grasping capability, trajectory stability, and user interaction performance, it does not fully reproduce the complexity of minimally invasive laparoscopic procedures. Future validation campaigns will incorporate more clinically representative tasks such as suturing, needle passing, tissue manipulation, and dissection.

Third, several sources of positioning error may affect overall system performance. These include calibration inaccuracies between the virtual and physical coordinate systems, communication latency between Unity, the C# application, and the robot controller, mechanical compliance of the robotic structure, assembly tolerances, actuator resolution limitations, and tracking uncertainties associated with the OptiTrack system.

Another important direction for future research involves the standardized performance evaluation of the proposed robotic platform according to internationally recognized robotic and medical-device standards, including ISO 9283 [55] and, where applicable, IEC 80601-2-77 [56]. Although such evaluations were beyond the scope of the present study, the current experimental setup provides the main components required for preliminary standardized characterization. The robot controller enables repeatable trajectory execution, while the external OptiTrack optical tracking system provides independent measurements of the robot end-effector without affecting the control loop. However, the present results do not demonstrate formal compliance with these standards or clinical readiness. Future investigations will therefore include standardized test poses and trajectories for assessing positioning accuracy, repeatability, path accuracy, path repeatability, trajectory deviation, motion smoothness, and positioning stability. In addition, the uncertainty of the OptiTrack-based measurement chain will be quantified through calibration residuals, repeated static and dynamic measurements, coordinate-frame registration analysis, and, where possible, comparison with reference measurement devices. This uncertainty budget will be reported together with the robot performance metrics in order to determine whether the measurement system is adequate for the required accuracy and precision assessment.

The AI-assisted module also presents limitations. Although the proposed DeepMLP–Vanilla LSTM ensemble achieved high predictive performance (R^2^ = 0.98801), the training dataset consisted of 2000 samples collected within a controlled experimental setup. Additional datasets acquired from a larger number of users, different manipulation tasks, and multiple robot configurations will be required to further improve model robustness and generalization capability.

Compared with conventional VR-based surgical training systems reported in the literature, the proposed framework introduces a tighter integration between the Digital Twin, the physical robotic platform, AI-assisted motion refinement, and independent optical motion validation. Nevertheless, additional comparative studies involving established laparoscopic simulators are required before definitive conclusions regarding training effectiveness can be drawn. practice.

Overall, the proposed framework represents a step toward the integration of immersive interaction, AI-assisted control, and physical robotic execution within a Digital Twin paradigm. The combination of VR-based control, external motion validation, and data-driven assistance provides a promising basis for future adaptive training environments and intelligent robotic systems for medical applications.

### 4.4. Statistical Analysis and Study Limitations

To further assess the experimental findings, a descriptive statistical analysis of the task completion times was performed. The analysis focused on comparing the overall performance trends observed under conventional operation and AI-assisted operation. Since the primary objective of the study was to evaluate the feasibility of integrating AI assistance within the proposed Digital Twin framework, task completion time was selected as the main performance indicator.

Due to the limited number of participants involved in this preliminary study (*n* = 6), the obtained results should be interpreted as an initial feasibility assessment rather than definitive evidence of performance superiority. Although the AI-assisted condition was associated with shorter task completion times, the sample size does not allow strong statistical conclusions regarding general performance improvements.

The results nevertheless suggest that the proposed AI-assisted Digital Twin framework has the potential to improve interaction efficiency while preserving the human-in-the-loop control paradigm. Future studies will include a larger participant cohort and a broader range of evaluation metrics, including trajectory smoothness, path efficiency, positioning accuracy, motion repeatability, synchronization latency, workload assessment, and user experience indicators. Such analyses will enable a more comprehensive statistical validation of the proposed approach.

## 5. Summary and Conclusions

The present work introduced a Digital Twin–based framework integrating immersive virtual reality interaction, real-time synchronization with the ATHENA parallel surgical robot, AI-assisted trajectory refinement, and independent OptiTrack-based motion validation. The proposed architecture demonstrated the feasibility of combining these technologies within a unified surgical robotics framework for robot interaction, training, and performance assessment.

Unlike conventional VR simulators, the proposed system establishes real-time correspondence between the virtual environment and the physical ATHENA robotic platform, allowing user-generated commands to be executed by the physical robot while maintaining synchronization with its Digital Twin representation. Furthermore, the integration of OptiTrack-based motion capture provides an independent mechanism for validating the correspondence between virtual and physical robot motion.

Experimental evaluation indicated promising results regarding interaction quality and task execution efficiency. However, the current validation involved a limited number of participants and a simplified manipulation task. Consequently, the reported findings should be interpreted as preliminary evidence supporting the feasibility of the proposed approach rather than definitive proof of performance improvement.

Future work will focus on larger-scale experimental studies, clinically relevant surgical tasks, advanced dynamic performance metrics, trajectory smoothness analysis, positioning accuracy assessment, workload evaluation, and enhanced Digital Twin synchronization strategies. Additional investigations will also explore the integration of advanced sensing technologies and intelligent assistance methods to further improve the effectiveness of Digital Twin–based surgical robotic systems.

## Figures and Tables

**Table 1:** Comparison of Surgical Robotic Systems and the Proposed AI Digital Twin Platform.

Ref.	VR Interaction	Digital Twin	Bidirectional Synchronization	Physical Robot	AI Assistance	External Validation	Surgical Application
[9]	Yes	Yes	Yes	Yes	No	Yes	Yes
[10]	Yes	Yes	No	Yes	No	Yes	Yes
[11]	No	No	No	Yes	Yes	Yes	Yes
[13]	Yes	No	No	No	No	Yes	Yes
[15]	Yes	No	No	No	No	No	Yes
Proposed work	Yes	Yes	Yes	Yes	Yes	Yes	Yes

**Table 2:** Input data structure.

Variable	Variable Semnification	Type	Minimum	Maximum	Mean
q_1	Coordinate of active articulation 1 of the parallel mechanism	input	0.00079	0.99770	0.49205
q_2	Coordinate of active articulation 2 of the parallel mechanism	input	0.00178	0.99943	0.49047
q_3	Coordinate of active articulation 3 of the parallel mechanism	input	0.00078	0.99879	0.49750
f_x	Movement the flexible tube on the *X* axis	input	0.00124	0.99871	0.49281
f_y	Movement the flexible tube on the *Y* axis	input	0.00343	0.99989	0.50575
r_f	Rotation of the surgical forceps around its own longitudinal axis	input	0.00060	0.99992	0.51120
s_1	Opening/closing angle of the first branch of the surgical forceps	input	0.00012	0.99699	0.50094
s_2	Opening/closing angle of the second branch of the surgical forceps	input	0.00140	0.99914	0.49424
t_x	Coordinate of the center point of the surgical forceps on the X axis	input	0.00051	0.99929	0.49901
t_y	Coordinate of the center point of the surgical forceps on the Y axis	input	0.00048	0.99889	0.50907
t_z	Coordinate of the center point of the surgical forceps on the Z axis	input	0.00014	0.99967	0.50166
b_v	Grip state of the blue cube	input	0	1	0.50356
r_v	Grip state of the red cube	input	0	1	0.48453
y_v	Grip state of the yellow cube	input	0	1	0.49287
b_c	Correct positioning status of the blue cube in the target area	input	0	1	0.51033
r_c	Correct positioning status of the red cube in the target area	input	0	1	0.50014
y_c	Correct positioning status of the yellow cube in the target area	input	0	1	0.50494

**Table 3:** Used hyperparameters.

Hyperparameter	Search Domain	Model	Selected Value
Epochs	{100, 200, 300, 400, 500, 600, 700, 800, 900, 1000}	DenseMLP	100
		DropoutMLP	1000
		BatchNormMLP	200
		DeepMLP	500
		WideMLP	500
		Vanilla LSTM	200
		Stacked LSTM	300
		Bidirectional LSTM	200
Learning rate	{0.0001, 0.0005, 0.001, 0.0015, 0.002}	DenseMLP	0.0005
		DropoutMLP	0.0001
		BatchNormMLP	0.0005
		DeepMLP	0.001
		WideMLP	0.0005
		Vanilla LSTM	0.001
		Stacked LSTM	0.0005
		Bidirectional LSTM	0.0015
Layers	{3, 4, 5, 6}	DenseMLP	3
		DropoutMLP	4
		BatchNormMLP	5
	{3, 4}	Stacked LSTM	3
	{1, 2}	Bidirectional LSTM	2
		WideMLP	2
	{8, 9, 10}	DeepMLP	9
Units	{16, 32, 64}	DenseMLP	32
		DropoutMLP	32
		BatchNormMLP	64
		DeepMLP	64
		Vanilla LSTM	32
		Stacked LSTM	32
		Bidirectional LSTM	32
	{512, 1024, 2048}	WideMLP	1024
Dropout rate	{0.1, 0.15, 0.2, 0.25, 0.3}	DropoutMLP	0.2

**Table 4:** Metric values.

Phase	Model	MAE	MdAE	RSME	R^2^
Train	DenseMLP	0.02765	0.02347	0.03589	0.98457
	DroupoutMLP	0.04885	0.04368	0.06065	0.95509
	BatchNormMLP	0.03299	0.02784	0.04198	0.97848
	DeepMLP	0.01858	0.01673	0.02318	0.99339
	WideMLP	0.02941	0.02501	0.03841	0.98229
	Vanilla LSTM	0.01898	0.01595	0.02626	0.99189
	Stacked LSTM	0.02626	0.02226	0.03456	0.98577
	Bidirectional LSTM	0.02801	0.02345	0.03683	0.98375
	Average Voting Ensemble	0.01192	0.01017	0.01611	0.99693
Validation	DenseMLP	0.03198	0.02713	0.04176	0.97874
	DroupoutMLP	0.05167	0.04491	0.06453	0.94821
	BatchNormMLP	0.05156	0.04206	0.06574	0.94622
	DeepMLP	0.03369	0.02939	0.04456	0.97591
	WideMLP	0.03096	0.02661	0.04045	0.97994
	Vanilla LSTM	0.02496	0.02081	0.03484	0.98545
	Stacked LSTM	0.02874	0.02487	0.03725	0.98315
	Bidirectional LSTM	0.02789	0.02319	0.03721	0.98321
	Average Voting Ensemble	0.02284	0.01932	0.03159	0.98801

## Data Availability

The original contributions presented in this study are included in the article. Further inquiries can be directed to the corresponding author.
